# Visual Telerehabilitation with Visually Impaired Children: From the Pandemic Emergency to a Stand-Alone Method

**DOI:** 10.3390/life13030725

**Published:** 2023-03-08

**Authors:** Giulia Perasso, Chiara Baghino, Elena Cocchi, Silvia Dini, Antonella Panizzi, Valentina Salvagno, Margherita Santarello, Aldo Vagge

**Affiliations:** 1 School of Life Cycle Psychology, University of Milano-Bicocca, 20126 Milan, Italy; 2 David Chiossone Foundation, 16123 Genoa, Italy; 3 Dipartimento di Neuroscienze, Riabilitazione, Oftalmologia, Genetica e Scienze Materno-Infantili, (DiNOGMI), Università degli Studi di Genova, 16126 Genoa, Italy; 4 IRCCS Ospedale Policlinico San Martino, 16132 Genova, Italy

**Keywords:** children, visual impairment, rehabilitation, telerehabilitation, orthoptics, orthoptist

## Abstract

In the last two years, orthoptists have counteracted patient drop-out through visual telerehabilitation. Efforts were made to transfer the in-person visual rehabilitation setting to the telematic environment in response to the worldwide crisis. Nowadays, statistical evidence on the effects of visual telerehabilitation is still scarce. The present research is the first, in Italy, to offer a pre-post assessment of the impact of visual telerehabilitation. Twenty-four (n = 24) children (64% male, 14% monocles) aged 4 to 15 years (mean age = 9.21 years, SD = 3.36, mean residual vision 1.3/10) were randomly assigned to three different group types for rehabilitation: a telematic rehabilitation group (n = 7), a mixed rehabilitation group (n = 8), and an in-person rehabilitation group (n = 9). Each group underwent a six-week visual rehabilitation. Ergo-perimetric evaluation before and after the rehabilitation was administered to the three groups. *t*-tests showed a significant improvement in ergo-perimetric outcomes in the visual telerehabilitation group (*p* < 0.05) and in the mixed rehabilitation group (*p* < 0.01), via a shortening of the response times. The findings suggest that visual telerehabilitation and mixed rehabilitation can lead to an ergo-perimetric improvement in visually impaired children within six weeks. Further research is needed, both to corroborate the findings with a larger sample size and to attain a follow-up measurement in order to clarify whether visual telerehabilitation could represent a stand-alone method.

## 1. Introduction

Visual rehabilitation has become an increasingly important aspect of healthcare for patients with visual impairment. According to existing scientific literature, visual rehabilitation is a fundamental approach to preventing vision loss and empowering visually impaired patients [1,2]. The importance of visual rehabilitation cannot be overstated. With the help of visual rehabilitation, visually impaired individuals can learn to adapt to their visual impairment and develop new strategies for navigating their daily lives. As a result, they can achieve a greater sense of autonomy and independence, leading to an improved quality of life. It is therefore essential to continue advancing the field of visual rehabilitation in order to provide individuals with the best possible care and support [1,2], since visual rehabilitation can foster social inclusion and empowerment among visually impaired patients [2]. By providing individuals with the skills and resources they need to navigate their environment confidently, visual rehabilitation can reduce the social isolation often experienced by visually impaired individuals. This, in turn, can enhance their psychosocial well-being and promote a greater sense of independence and self-efficacy.

Visual rehabilitation protocols have been designed to address a wide range of visual impairments and enhance various aspects of visual function. For instance, visual rehabilitation can improve synaptic responses and cortical plasticity [3,4], which are essential neural mechanisms underlying visual perception and cognition. These improvements can be achieved through a variety of techniques, including visual stimulation, task-specific training, and perceptual learning. In addition to enhancing neural plasticity, visual rehabilitation can also improve the functional abilities of visually impaired patients through the training of compensatory eye movements [5], visual attention [6], and eccentric fixation [7]. These techniques aim to optimize the use of residual vision and compensate for visual deficits, thereby improving visual function and overall quality of life.

The COVID-19 pandemic has posed significant challenges to the global healthcare system. One of the major challenges has been the temporary disruption of healthcare services, including rehabilitation services. Additionally, restrictive anti-contagion measures have increased the risk of rehabilitation drop-out for individuals with disabilities [8]. In order to overcome these challenges, professionals in various fields have adapted rehabilitation protocols to the telematic environment [9,10,11,12]. However, the adaptation of visual rehabilitation protocols was particularly challenging, due to a lack of international guidelines, and the difficulties faced by visually impaired patients in using technology [13,14,15]. Despite these difficulties, rehabilitators responded to the COVID-19 emergency by structuring telerehabilitation programs to treat visually impaired patients via Internet Communication Technology (ICTs) devices such as personal computers and tablets [11]. Recent literature has highlighted the importance of the therapeutic alliance between the patient and the orthoptist, as well as the duration of the sessions (usually 30–40 min), for successfully conducting visual telerehabilitation, especially for child patients [16,17].

Nevertheless, there is still limited evidence on the effects of visual telerehabilitation. This approach was implemented in response to the COVID-19 emergency, without specific a priori research designs. Thus, the present study aims to measure the effects of visual telerehabilitation, alongside mixed rehabilitation (i.e., integrating online and in-person sessions), and traditional in-person visual rehabilitation over a six-week period. The study was conducted in Italy and is one of the first to explore the potential of telematic services for children with visual impairment.

## 2. Materials and Methods

### 2.1. Participants

Children in rehabilitation at David Chiossone Foundation (Genoa, Italy) took part in the research between January and May 2021, after the administration of informed consent for participation to their parents. Children were included in the study if: (i) their age was between 4 and 16 years, (ii) they exhibited residual vision between 1/10 and 3/10, (iii) they had monocular or binocular vision, (iv) they exhibited diagnosis determining a visual impairment. Exclusion criteria applied in order to constitute the sample were as follows: (i) age above 16 or below 4, (ii) vision inferior to 1/10 and above 3/10, (iii) they had multiple disabilities. Twenty-four (n = 24) children (64% male, 14% monocles) aged 4 to 15 years (mean age = 9.21 years, SD = 3.36) constituted the sample of the research. The children exhibited a wide range of pathologies determining the visual impairment (e.g., nystagmus, Sickler syndrome, congenital cataract, retinoblastoma, Leber atrophy, ocular albinism, optic glioma, retinopathy, strabismus, microphthalmos, glaucoma, ceroid lipofuscinosis, encephalocele, and optic atrophy in osteopetrosis). The mean residual vision of the sample was 1.3/10, in a range between 1/10 and 3/10. Children were randomly assigned to three different types of rehabilitation: (i) a telematic rehabilitation group comprising n = 7 children; (ii) a mixed-rehabilitation group comprising n = 8 children; (iii) and an in-person rehabilitation group comprising 9 children.

### 2.2. Telematic Visual Rehabilitation

Visual telerehabilitation consisted of six online sessions (one per week) of 30–40 min each, conducted by an orthoptist. Each session presented exercises stored in folders on Microsoft Teams, classified by program (e.g., Microsoft PowerPoint or Microsoft Word), session number, children’s age, and specific features (e.g., numbers, pictures, and sounds). The exercises aimed at training different visual abilities [17]: (a) fixation stability: the capacity to keep the gaze on a target; (b) visual pursuit: the capacity to gaze-follow objects in motion; (c) visual search and exploration: the ability to search for a target that disappears and reappears in different positions; (d) saccadic eye movements: the ability to shift the gaze from one target to another; (e) visual attention: the capacity to detect details in a changing scene; (f) visual-spatial-motor coordination: the capacity to detect the spatial orientation of an object. With all of these visual abilities stimulated, visual telerehabilitation aims to improve overall visual functioning in VI children. Each online session took place on Microsoft Teams; the orthoptist and the child were in a video call, with the orthoptist sharing their screen or showing PowerPoint presentations to the child.

Before beginning visual telerehabilitation, the orthoptists had to provide VI children’s parents with the technical and pragmatic instructions to constitute the best setting in which to practice the telerehabilitation session. Parents were instructed to: (i) prepare a silent room, without sensory stimuli that could distract the child; (ii) position the child on a chair in front of a desk, on which the screen (computer or tablet) was positioned; (iii) not hold the child during the sessions, (iv) keep the webcam in a position where it could capture the eye movements of the child. Eventually, parents were also instructed to print materials for the exercises. On the other hand, the orthoptist conducted the online sessions in a setting that constituted of a desk station with a personal computer, accessorized with a webcam, a microphone, speakers, and an Internet connection.

### 2.3. Measures

**Figure 1:**
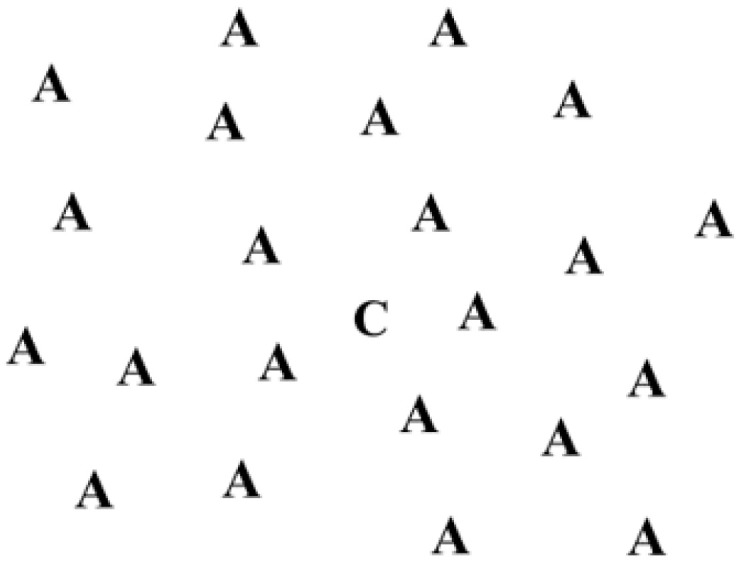
The target is in the center of the screen.

**Figure 2:**
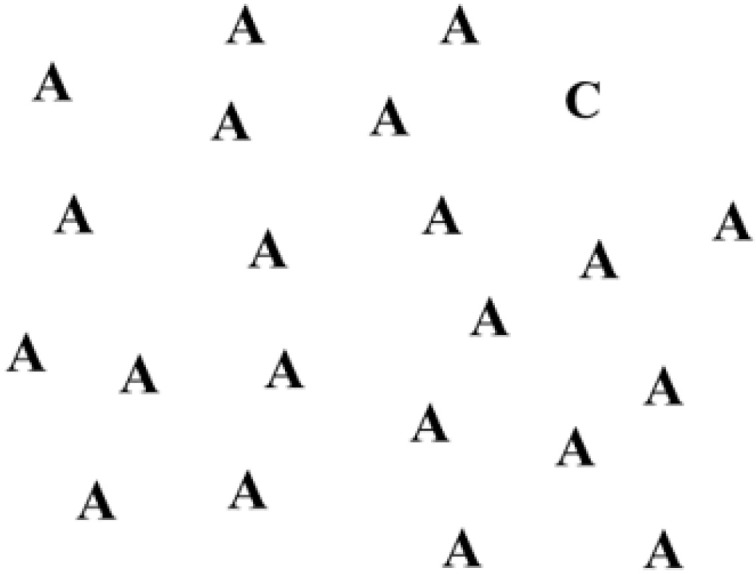
The target appears in another position.

In the present research, the outcome utilized to evaluate the impact of the three types of rehabilitation was the response time of patients at the ergo-perimetric assessment. Ergo-perimetry is an assessment of the visual response of the patient, used to monitor the outcomes of rehabilitation [17]. To practice ergo-perimetry, orthoptists require a semi-dark setting, where black-and-white slides are projected on an empty wall (i.e., creating projections of 2 × 1.5 m, in an area of 30° of eccentricity) [18,19]. Each slide shows standardized stimuli (e.g., shapes, playing cards, letters, numbers, or words). One of the stimuli (i.e., target) is kinetic and it changes position with respect to the other static stimuli scattered in the background of the picture. In the first shown slide (Figure 1), the target overlaps the patient’s fixation point. In the second slide, it disappears from the center and reappears at other points in the space (Figure 2).

The patient has the task to gaze, identify, and verbally account for the position of the target while the orthoptist measures the patient’s response time with a chronometer. The response times of all 3 groups of participants were assessed with this procedure 1 week before and 1 week after the rehabilitation.

### 2.4. Analytic Plan

In order to determine if there were any statistically significant changes in the reaction times observed at ergo-perimetry as a result of the three types of rehabilitation, means for all three groups were analyzed and compared before and after the rehabilitation programs. To achieve this objective, a one-sample *t*-test was employed for each group, which allowed for paired observations to be compared with the objective of identifying any statistically significant differences between pre- (t1) and post (t2) rehabilitation. The results of these analyses are presented in Table 1.

## 3. Results

In the telematic visual rehabilitation group, a statistically significant improvement (*p* < 0.05) was observed between the pre- and post-rehabilitation means at ergo-perimetry, indicating a reduction in response times (Table 2). The effect size was found to be large in this group, with Cohen’s d = 1.16, suggesting a substantial impact from the intervention. Similarly, in the mixed rehabilitation group, a statistically significant improvement (*p* < 0.01) was observed between the pre- and post-rehabilitation means at ergo-perimetry, indicating a reduction in response times (Table 2). The effect size was found to be large in this group as well, with Cohen’s d = 0.15, although it was relatively smaller than that observed in the telematic rehabilitation group. In contrast, the group that underwent in-person rehabilitation did not exhibit any statistically significant differences between pre- and post-rehabilitation means at ergo-perimetry (Table 2). These results suggest that telematic and mixed rehabilitation approaches may be more effective than in-person rehabilitation for improving response times at ergo-perimetry.

## 4. Discussion

After six weeks, the children provided with visual telerehabilitation, and those provided with mixed rehabilitation showed an improvement in their response times in the ergo-perimetry assessment. No amelioration emerged in the traditional rehabilitation group. As the children were randomly assigned to the three types of rehabilitation, it is possible to speculate that the in-person group did not show an improvement for different intervening variables that should be considered and statistically controlled in future studies (e.g., in the child: medical conditions, cognitive functioning, motivation, etc.; in the orthoptist: motivation, specific goals tailored to the patients, etc.).

The use of technology in healthcare has revolutionized the way medical treatments are delivered to patients [20]. Visual rehabilitation is an area of healthcare that has greatly benefited from the use of technology. The use of visual telerehabilitation has been shown to improve the ergo-perimetric response of visually impaired children in a limited time of six weeks. This finding is particularly noteworthy because it shows that visual telerehabilitation has the potential to improve visual skills during the developmental age. The positive outcome observed in this study for the visual telerehabilitation and mixed rehabilitation groups could be attributed to the ease of engagement that children of the current generation have with technology and ICT devices.

This study’s findings also suggest that visual telerehabilitation could be a stand-alone method in the field of visual rehabilitation [21]. This could potentially save patients and their families the efforts and costs of traveling to clinics that provide the service. In addition, it could help them overcome geographical and physical barriers that limit access to visual rehabilitation services [22]. The findings of this study are consistent with a recent scoping review that stated that telerehabilitation for visually impaired patients can provide a wide range of positive benefits to individual quality of life [20]. However, further studies in the field are needed, since limited real-world data on the long-term effects of telerehabilitation for people with low vision are available. This study is significant because it is the first to statistically test the effects of visual telerehabilitation [9,10,11,12,16,17], and it has been able to catalyze and systemize the efforts of orthoptists who conducted pioneering visual telerehabilitation interventions during the pandemic [16,17].

Despite the promising findings of this study, its small sample size constitutes a limit. Methodologically, small sample sizes can lead to less precise statistical estimations [23]; a future direction in this investigation should be to include a greater number of individuals in the three groups of participants, in order to achieve a major generalizability of the results. Another limitation that should be addressed by future studies on visual telerehabilitation for children is constituted by the lack of a follow-up assessment to investigate whether and how long the improvement observed in the study remains in the long term; as highlighted in the literature [24], a follow-up assessment, at least six months after the intervention, is essential for assessing the duration of the results and to monitor the intervention’s validity over time. A final limitation of this study is the use of only an ergo-perimetry assessment as the outcome variable. Future research should also include other measures alongside ergo-perimetry in order to test the impact of the three different types of visual rehabilitation on the patients. In fact, using multiple measures can help to address other existing issues and provide a more comprehensive understanding of the effects of the intervention. Future studies in this direction should include other measures, alongside the ergo-perimetry, to increase the reliability of the findings and reduce the impact of threats to internal validity [25]. In conclusion, the use of visual telerehabilitation in the rehabilitation of visually impaired children has been shown to improve visual skills, even in a limited time of six weeks. The potential benefits of visual telerehabilitation include overcoming geographical and physical barriers, reducing the effort and cost of traveling to clinics, and improving access to visual rehabilitation services. In this field, the study results provide a starting point for further research on the use of visual telerehabilitation as a stand-alone method.

## 5. Conclusions

This study represents the first research in Italy to examine the potential of visual telerehabilitation in improving the visual function of children with visual impairment (VI). By investigating the feasibility and effectiveness of this telemedicine-based approach, this study served to catalyze the pioneering efforts of orthoptists during the COVID-19 pandemic. The findings of the study suggested that visual telerehabilitation could be a promising tool for improving the visual function of VI children, as it allows the training of compensatory eye movements, visual attention, and eccentric fixation.

The results of this study also indicate that visual telerehabilitation could be a valuable means of providing access to vision rehabilitation services for VI children and their families, particularly in situations where physical distancing and lockdowns are required due to infectious disease outbreaks such as COVID-19. As a stand-alone method, visual telerehabilitation has the potential to overcome several barriers in the care of VI children, including physical distance, limited availability of specialized services, and contagion risks.

Overall, the findings of this study lay the groundwork for further research and testing of the effects of telematic healthcare and rehabilitation services for VI children. By exploring the feasibility and effectiveness of visual telerehabilitation in improving visual function, this study contributes to the growing body of literature on the use of telemedicine-based approaches for visual rehabilitation in VI patients. Future studies should focus on testing the long-term effects of visual telerehabilitation, as well as exploring other measures of visual function beyond ergo-perimetry. Such efforts could help to develop more comprehensive and effective telemedicine-based approaches for the care of VI patients, from the developmental age.

## Figures and Tables

**Table 1:** Descriptive statistics: means pre- and post-rehabilitation of the 3 groups.

		Mean (Seconds)	N	SD
Telematic	Ergo-perimetry t1	0.85	7	0.54
	Ergo-perimetry t2	0.42	7	0.45
Mixed	Ergo-perimetry t1	0.97	8	0.75
	Ergo-perimetry t2	0.79	8	0.66
In person	Ergo-perimetry t1	0.44	9	0.29
	Ergo-perimetry t2	1.30	9	1.84

**Table 2:** One sample *t*-test for paired observation.

		Mean (Seconds)	SD	St. Error Mean	95% Confidence Interval of the Difference		t	df	Sig. (2-tails)
					**Lower**	**Upper**			
**Telematic**	t1–t2	0.43	0.38	0.14	0.08	0.78	3.01	6	0.024 *
**Mixed**	t1–t2	0.17	0.12	0.04	0.07	0.28	4.02	7	0.005 **
**In person**	t1–t2	−0.86	1.76	0.59	−2.21	0.49	−1.47	8	0.179

* Statistically significant at *p* < 0.05, ** Statistically significant at *p* < 0.01.

## Data Availability

Not applicable.
